# Research on Quantitative Detection of Industrial Mixed Gases Based on Improved BP Neural Network

**DOI:** 10.3390/s26103100

**Published:** 2026-05-14

**Authors:** Xudong Shen, Jianping Zhu, Tian Tian

**Affiliations:** 1 College of Engineering Science and Technology, Shanghai Ocean University, Shanghai 201306, China; 15168251278@163.com; 2 School of Integrated Circuits, Shanghai Jiao Tong University, Shanghai 200240, China; tian1997@sjtu.edu.cn

**Keywords:** mixed gas detection, sensor array, BP neural network, Sledge Dog Optimizer (SDO), cross-sensitivity

## Abstract

To address the cross-sensitivity and non-linear coupling issues caused by the coexistence of hydrogen, carbon monoxide, ammonia, and nitrogen dioxide in industrial environments, a flow-through quantitative detection system based on a MEMS gas sensor array was designed and constructed. The steady-state peak sampling method was employed for feature extraction from high-dimensional time-series data, and regression prediction models were developed using a traditional BP neural network and BP neural networks optimized by four swarm intelligence algorithms (ALA, AOO, SFOA, and SDO). The experimental results indicate that the intelligent optimization algorithms excel in decoupling the “cross-response” phenomenon, with all optimized models outperforming the traditional BP network. Among them, the SDOBP (Sledge Dog Optimizer-BP) model demonstrated the best overall performance, achieving the highest accuracy in carbon monoxide and hydrogen detection, with the Root Mean Square Error for hydrogen reduced to 2.17, an 84.2% improvement over the traditional model. The system achieves high-precision quantitative inversion of multi-component gases in complex environments, providing an effective means for industrial environmental safety monitoring.

## 1. Introduction

In recent years, with the global industrial transition toward low-carbon and intelligent systems, the safety risks posed by the coexistence of multi-component gases in complex operational environments have become increasingly prominent [1]. In fields such as energy and chemical engineering, power monitoring, and underground engineering, the real-time monitoring of key characteristic components—including hydrogen (H_2_), carbon monoxide (CO), ammonia (NH_3_), and nitrogen dioxide (NO_2_)—is critical for system safety assessment. However, these gases often exist in a coupled state in practical environments. The cross-sensitivity between sensors makes it difficult for single detection methods to achieve accurate quantitative analysis. To address this cross-sensitivity issue, Acharyya S. et al. [2] constructed a sensor array by decorating SnO_2_ hollow spheres with Ag, Au, Pd, and Pt nanoparticles. By integrating a deep neural network model based on time series (DNN_TS), they achieved precise quantitative prediction of various VOCs, with an average concentration prediction error as low as 4.75%. Zbigniew S. et al. [3] developed an electronic nose system comprising 17 metal oxide semiconductor (MOS) sensors. By combining Support Vector Machine (SVM) and Multi-Layer Perceptron (MLP) algorithms, they realized the precise prediction of eight fungal metabolite markers in indoor air. Among these, the SVM model exhibited superior quantitative performance, with an average coefficient of determination (*R*^2^) reaching 0.975 and a Root Mean Square Error (*RMSE*) of only 0.004. Furthermore, Li et al. [4] utilized four types of semiconductor gas-sensing materials (In_2_O_3_, Pd-ZnO, Au-SnO_2_, and Pd-LaFeO_3_) to construct a sensor array. They proposed a Local Dynamic Neural Network (LDNN) model combined with pre-trained Auto-Encoder (AEN) feature extraction, achieving precise prediction for a mixture of NO_2_, NH_3_, CH_4_, and CO_2_, with all Mean Relative Errors (MREs) maintained within 1.17%.

Existing research has predominantly focused on a single category of gases (such as VOCs or NO_x_ systems), while systematic studies targeting the specific gas combination of H_2_–CO–NH_3_–NO_2_ remain relatively scarce. Furthermore, issues such as the interference effects between different gas species, the optimal design of sensor arrays, and robust feature extraction methods continue to be significant challenges in current research.

To address the aforementioned issues, this paper designs and constructs a multi-component gas sensor array comprising hydrogen, carbon monoxide, ammonia, and nitrogen dioxide sensors, aiming to achieve simultaneous detection and differentiation of multiple key gases in typical industrial environments. By integrating pattern recognition and machine learning algorithms, the system performs feature extraction and quantitative identification of multiple gases through the analysis of response signals from each sensor within the array.

## 2. Materials and Methods

### 2.1. Overview of Quantitative Detection of Mixed Gases

In complex industrial sites, the components of mixed gases often exhibit highly nonlinear coupling characteristics. Due to the limitations of cross-sensitivity, traditional single-sensor detection modes struggle to accurately decouple individual gas concentration information from overlapping response signals [5]. This study adopts a collaborative detection scheme based on a sensor array. The core concept is to leverage the response variations of different sensors within the array to target gases—such as hydrogen, carbon monoxide, ammonia, and nitrogen dioxide—to construct a multi-dimensional “electronic olfaction” space. Through subsequent signal processing and regression algorithms, the physicochemical responses of the sensors are transformed into quantitative concentration values for each component. The fundamental detection workflow can be summarized as gas sample preparation, sensor array configuration, signal processing module, regression prediction module, and final gas concentration output.

The core of quantitative analysis for mixed gases lies in leveraging regression prediction algorithms to deeply mine the intrinsic correlations within multi-dimensional sensing data, thereby constructing an accurate concentration mapping model [6]. Experimental research reveals that the response characteristics of the sensor array exhibit distinct stage-based features: in the low-concentration range, the output voltage tends to be linearly correlated with the gas components; however, as concentrations increase and multi-component cross-interference intensifies, the system demonstrates significant nonlinear coupling and physical saturation characteristics. To precisely capture this complex mapping relationship, this study adopts the steady-state peak sampling method [7]. Key voltage points are extracted at characteristic moments when the sensor response reaches dynamic equilibrium, effectively reducing high-dimensional time-series data into feature vectors that represent the gas information.

### 2.2. Sensor Selection

All sensors listed in Table 1 are Micro-Electro-Mechanical Systems (MEMS) gas sensors. Based on their structural design, they are categorized into four-pin and ceramic-packaged sensors. While the current sensor array utilizes established MEMS technology, it is important to acknowledge that emerging low-dimensional materials, such as InSe-based sensors, represent a significant frontier in hardware sensitivity. For instance, Au-decorated InSe sensors have demonstrated high-sensitivity and UV-free detection at room temperature [8], and integrated InSe-based systems have achieved the simultaneous monitoring of NO_2_ gas and temperature with high selectivity [9]. Although these next-generation 2D materials are not integrated into the present prototype, the proposed SDO-BP algorithm provides a universal and scalable intelligent framework. It is designed to be potentially coupled with such high-performance sensing units in future iterations to further enhance detection limits and selectivity in complex industrial environments. The circuit schematic integrates the sensors and the power supply circuitry onto a single Printed Circuit Board (PCB).

### 2.3. System Architecture and Operational Workflow for Mixed Gas Detection

Since the gas sensing process is essentially a sequence of adsorption, diffusion, and charge exchange of gas molecules on the surface of sensitive materials, this mechanism renders the sensor’s response highly sensitive to the concentration distribution and flow state, a phenomenon known as flow-field dependency [10]. To eliminate interference from environmental fluctuations and obtain stable, reproducible test samples, this study eschews the static gas distribution method, which is prone to uneven local concentration gradients. Instead, a flow-through detection scheme [11,12] is adopted, offering higher precision and more constant mass transfer conditions.

**Figure 1:**
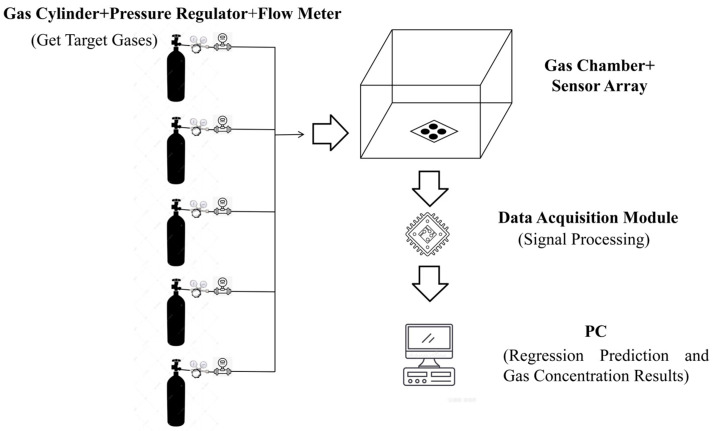
Operational schematic of the integrated data acquisition and gas sensing system.

**Figure 2:**
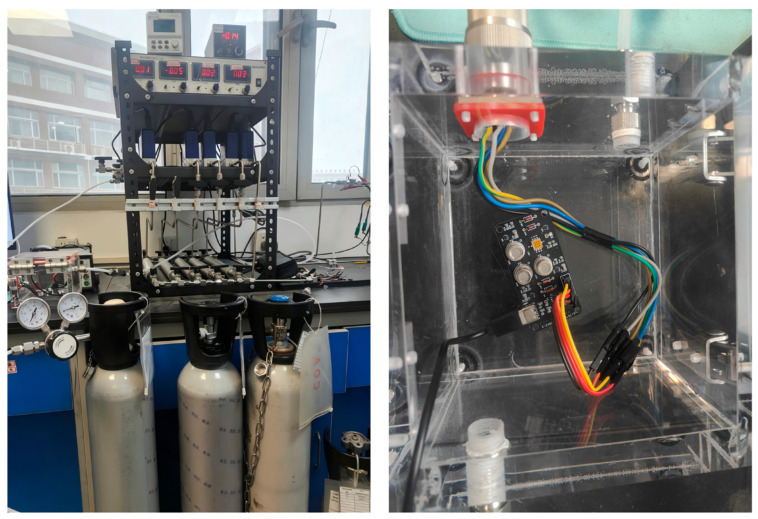
Physical photograph of the experimental prototype and experimental setup.

To meet the aforementioned testing requirements, this study implemented a specialized integrated design for the peripheral gas pathways and the gas chamber structure of the acquisition system. A miniaturized gas chamber with a compact volume and excellent airtightness was designed to further reduce response latency and enhance detection sensitivity [13]. The layout of the sensor array was strictly optimized based on the chemical properties and concentration ranges of the target gases to maximize the capture of cross-response characteristics of the multi-component mixture. To obtain the data required for this research, a self-designed acquisition system was employed, featuring a sensor array integrated with four specific gas sensors (as detailed in Table 1). Within the hardware system, an STM32F103C6T6A microcontroller (STMicroelectronics, Geneva, Switzerland) serves as the main control module. The sensor array within the chamber reacts with the target gases to generate electrical signals. The schematic diagram of the system operation is shown in Figure 1, and the physical photograph of the detection system is presented in Figure 2.

The operational workflow of the device is as follows:

(1) Device Initialization: The gas channels are first purged with ambient air for 1 min for cleaning, followed by a 3 min preheating period for the gas sensor detection system to ensure stability.

(2) Sampling and Detection: In this study, mixed gases of varying concentrations were prepared using a precision gas distribution instrument. Ambient air served as the diluent, while the source gases consisted of purchased standard-concentration H_2_, CO, NH_3_, and NO_2_. By simultaneously proportioning the diluent and source gases, seven concentration gradients were selected for each of the four gas species. A total of 96 groups of distinct mixed gas combinations were configured based on flow rate ratios for the experiments. Each detection cycle lasted 5 min and followed a specific sequence: 1 min of air intake (baseline), 3 min of mixed gas exposure (reaction), and a final 1 min of air purging (recovery).

(3) Signal Processing: The signals generated by the sensors are rectified and filtered by the sampling circuit. Subsequently, Analog-to-Digital (A/D) conversion is performed via the I/O ports of the microcontroller, converting the detected analog signals into digital data recognizable by the software. Finally, these digital signals are transmitted to a PC via USART serial communication for data storage and analysis.

In this study, although active compensation sensors were not integrated, the influence of moisture was suppressed through the hardware-level stabilization of the flow-through scheme. Specifically, the 1 min high-purity air purging phase at the start of each cycle effectively desorbs residual water molecules from the sensor surface, establishing a consistent initial baseline.

### 2.4. Sampling and Data Acquisition

In this study, the coexistence scenarios of four-component industrial gases were simulated using the dynamic gas distribution method. Ambient air was used as the diluent and proportioned with standard-concentration source gases, including hydrogen, carbon monoxide, ammonia, and nitrogen dioxide. To ensure both data diversity and experimental efficiency, the concentration gradients of the four gases were divided into seven levels according to orthogonal design principles. Ultimately, sensor response data for 96 groups of different concentration combinations were obtained [14]. To meet the verification requirements of machine learning algorithms, the experimental dataset was partitioned into two subsets: 84 groups were used as training samples, while the remaining 12 groups served as test samples to evaluate the predictive performance of the regression model. The specific concentration selection scheme is detailed in Table 2.

### 2.5. Data Processing and Analytical Methodology

In sensor signal processing, the quality of feature extraction directly determines the predictive accuracy of the subsequent regression model. Through an analysis of the dynamic response curves from the 96 experimental samples, this study found that after contact with the target gases, the charge exchange process tends toward equilibrium at the end of the gas-flow cycle. At this stage, the output voltage most accurately reflects the saturation level of gas molecules covering the surface of the sensitive material.

Based on this observation, the steady-state peak sampling method was adopted to extract key feature points from the continuous voltage sequences. Within each 5 min detection cycle, the final moment of the mixed gas exposure phase (at the 240th second of the sampling sequence) was selected as the feature extraction point. At this juncture, the sensor has completed 3 min of sufficient reaction, and the response voltage reaches the steady-state peak V_s_ for the given concentration. This voltage value represents the instantaneous sensing equilibrium of the sensor array in the environmental atmosphere under specific flow rates and mixing ratios.

Before performing regression prediction, data normalization [15] was applied to eliminate weight imbalances caused by differences in sensor ranges, ensuring that each sensor channel contributes equally during model training. Subsequently, the 96 groups of sample data were randomly partitioned into a training set and a test set at a ratio of 7:1. Finally, regression prediction models were constructed using a traditional BP Neural Network and four swarm intelligence optimization algorithms: Artificial Lemming Algorithm (ALA-BP), Animated Oat Optimization (AOO-BP), Superb Fairy-wren Optimization Algorithm (SFOA-BP), and Sledge Dog Optimizer (SDO-BP) [16,17,18,19].

The selection of ALA, AOO, SFOA, and SDO as optimization benchmarks is based on their unique theoretical architectures, which address the specific challenges of gas sensor array decoupling, such as high-dimensional non-linear coupling and cross-sensitivity. SDO was chosen for its robust global search capability and the “collaboration–retirement” mechanism, which is particularly effective for large-amplitude signals like H_2_ and CO detection; ALA is included due to its superior exploration–exploitation balance governed by the time-varying θ factor; AOO is selected for its high convergence speed; SFOA provides exceptional local exploitation through its sine-triggered search patterns.

The detailed configurations of the BP neural network and the specific parameters of the swarm intelligence algorithms are summarized in Table 3 and Table 4.

The BP neural network architecture consists of a single hidden layer with 9 neurons, determined by the empirical formula 2*n* + 1, where *n* is the number of input neurons. The logsig and purelin functions are employed as the transfer functions for the hidden and output layers, respectively, to map the complex nonlinear relationship between sensor responses and gas concentrations. The Levenberg–Marquardt (trainlm) algorithm is selected as the weight optimization method due to its efficient convergence.

For the swarm intelligence algorithms, all models operate within an 85-dimensional search space, representing the total number of weights and thresholds in the 4-9-4 network structure. To maintain a fair comparison, the maximum number of iterations is fixed at 20 for all optimizers. The exploration and exploitation phases are balanced through specific time-varying coefficients. For instance, the SDO-BP model utilizes a linearly decreasing inertia weight $\omega_{1}$ and a non-linear factor $p_{1}$ combined with chaotic perturbations to enhance global search capability and prevent the model from becoming trapped in local optima.

### 2.6. Performance Evaluation Metrics

To quantitatively evaluate the inversion accuracy and robustness of the neural network models optimized by swarm intelligence algorithms in mixed gas detection, three regression performance metrics are introduced in this study. By comparing the performance of the traditional BP neural network and the optimized models on the test set, the degree of deviation between the predicted concentrations and the actual concentrations is measured from various dimensions. The following three evaluation metrics were selected: Coefficient of Determination (*R*^2^), Root Mean Square Error (*RMSE*), and Mean Absolute Percentage Error (*MAPE*) [20].

(1) $$R^{2}=1-\frac{\sum_{i=1}^{n}(y_{i}-{\hat{y}}_{i}{)}^{2}}{\sum_{i=1}^{n}(y_{i}-\bar{y}{)}^{2}}$$

(2) $$RMSE=\sqrt{\frac{1}{n}\sum_{i=1}^{n}(y_{i}-{\hat{y}}_{i}{)}^{2}}$$

(3) $$MAPE=\frac{100\%}{n}\sum_{i=1}^{n}\frac{\left|y_{i}-{\hat{y}}_{i}\right|}{y_{i}}$$

where n is the number of samples, $y_{i}$ represents the actual observed gas concentration, ${\hat{y}}_{i}$ denotes the predicted value from the model, and $\bar{y}$ is the mean of the actual values. The value of *R*^2^ ranges between [0, 1]. A value closer to 1 indicates a more accurate construction of the mapping relationship between the sensor response signals and the gas concentrations, signifying a stronger capability of the model to decouple the complex nonlinearities of the mixed gases. A smaller *RMSE* suggests that the model possesses enhanced robustness when dealing with cross-sensitivity interference in mixed gases, resulting in lower volatility of the prediction results. A *MAPE* value closer to 0% demonstrates higher precision in the quantitative analysis of mixed gases across different measurement ranges.

## 3. Experimental Results and Discussion

### 3.1. Analysis of Sample Data

Table 5 presents a portion of the sample data collected by the acquisition system, where S1, S2, S3, and S4 correspond to the carbon monoxide, nitrogen dioxide, ammonia, and hydrogen sensors, respectively. As indicated by the output voltages of the sensor array in the table, there are significant discrepancies among the data points. The sensors within the array do not solely respond to their specific target gases; instead, a pronounced cross-sensitivity phenomenon is observed. For instance, comparing the data for Index 40 and Index 47, when the CO concentration increases from 500 ppm to 550 ppm, the output voltage of S1 (the CO sensor) changes as expected. However, simultaneously, the voltages of S2 (NO_2_ sensor), S3 (NH_3_ sensor), and S4 (H_2_ sensor) also rise from 3066 mV, 203 mV, and 943 mV to 3087 mV, 207 mV, and 949 mV, respectively, despite their corresponding gas concentrations remaining constant. This “one-gas–multiple-responses” phenomenon demonstrates severe signal coupling between the mixed gas components. Consequently, the output of a single sensor cannot accurately invert the concentration of a specific gas, which underscores the necessity of introducing swarm intelligence optimization algorithms for multi-dimensional decoupling in this study.

From an overall trend, the output voltages of the sensors exhibit a strong positive monotonic correlation with their corresponding target gas concentrations. Taking S2 (the NO_2_ sensor) as an example, as the NO_2_ concentration increases gradiently from 1 ppm to 5 ppm, its output voltage grows steadily from 2924 mV to 3411 mV. However, this growth is not a simple linear relationship; as the concentration steps up, the magnitude of the voltage increment gradually diminishes. This reflects the typical nonlinear characteristics of metal-oxide semiconductor (MOS) sensors, where the active sites on the surface of the sensitive material tend toward saturation when exposed to high gas concentrations.

S2 exhibits a high baseline output voltage (approximately 3000 mV) and is extremely sensitive to trace variations in NO_2_. In contrast, S1 shows a lower initial response voltage in low-concentration CO environments (around 160–170 mV) but demonstrates a larger dynamic range in high-concentration regions (e.g., Indices 91–95). These differentiated response characteristics provide the sensor array with rich feature information, making it possible to construct an accurate mapping from multi-dimensional voltage points to the multi-dimensional concentration space using complex algorithms such as SDO-BP.

### 3.2. Convergence Analysis of Optimization Algorithms

**Figure 3:**
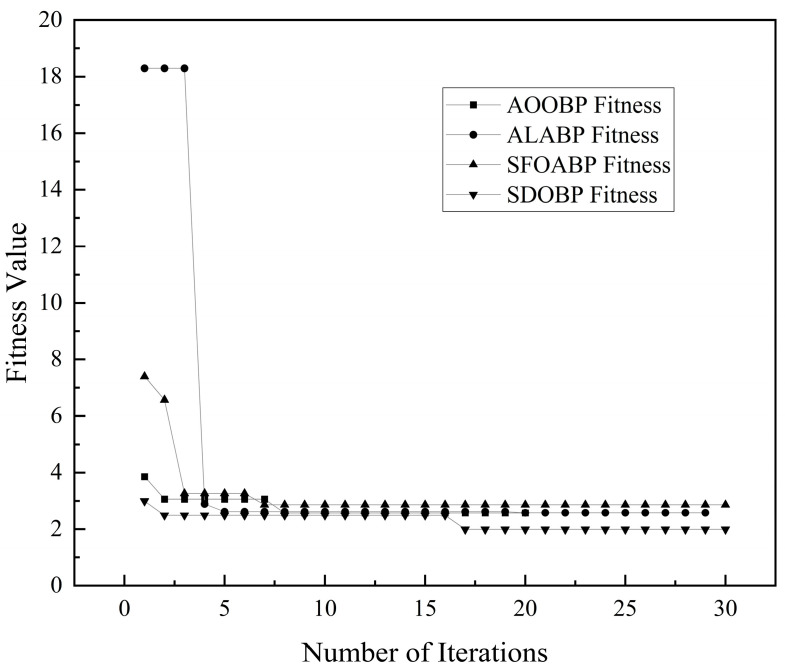
Iterative fitness curves for the compared BP neural network models.

The fitness curve of an algorithm serves as a core criterion for evaluating the optimization efficiency of swarm intelligence techniques [21]. As illustrated in Figure 3, ALA-BP and SFOA-BP exhibit high initial fitness values in the early stages (the first 5 iterations), followed by significant step-like declines. This indicates that these algorithms possess larger exploration step sizes during the initial phase, allowing them to rapidly migrate from random states toward high-potential regions [22]. AOO-BP reaches a stable plateau within a very small number of iterations, demonstrating a faster convergence rate. However, the performance of SDO-BP is the most prominent; its curve undergoes a secondary descent around the 15th to 17th iterations and ultimately settles at the lowest fitness level among all models. This proves that the SDO algorithm possesses a superior ability to escape local optima.

### 3.3. Comparison and Regression Analysis of Predicted and Actual Concentrations

**Figure 4:**
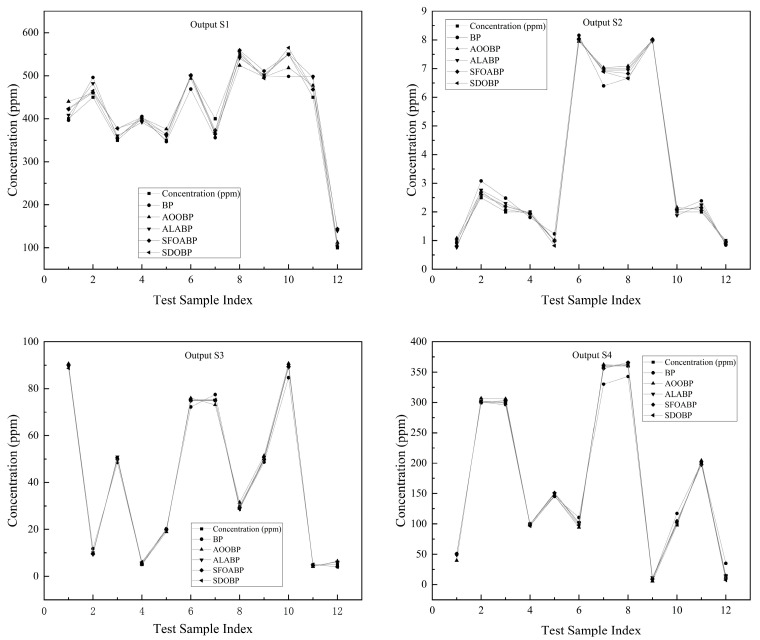
Comparison between predicted and actual gas concentrations on the test set.

Combined with the comparison curves of predicted versus actual values in Figure 4, the decoupling effectiveness of the SDO-BP model across different gas components can be intuitively observed. In the regression task for S1 (CO sensor), where concentration fluctuations are substantial, the prediction points of the traditional BP (represented by circles) exhibit significant deviations. In contrast, the trajectory of the SDO-BP model (left-pointing triangles) maintains a high degree of consistency with the actual values (squares). Notably, at the high-concentration peaks for samples 8 to 11, SDO-BP accurately captures the signal features without any noticeable peak clipping. Simultaneously, for the outputs of S2, S3, and S4, the predictive accuracy of all optimization algorithms generally surpasses that of the base BP network. Among them, SDO-BP demonstrates a near-perfect fit for the S4 output (H_2_ sensor), where the prediction points almost entirely overlap with the actual value curve.

### 3.4. Statistical Analysis of Performance Metrics

To address the bottlenecks of traditional BP neural networks in the quantitative inversion of mixed gases—such as slow convergence and the tendency to become trapped in local minima—this study introduces four optimization algorithms to optimize the weights and thresholds of the network. According to Table 6, by comparing the regression performance of the traditional BP model with that of the optimized models, the experimental results demonstrate that all four optimization algorithms achieved a comprehensive superiority over the traditional BP network across all output dimensions.

The *R*^2^ values of the traditional BP neural network across all dimensions ranged between 0.93 and 0.99, while the optimized models consistently outperformed the traditional BP network in every sensor output. This indicates that the proposed algorithms can more accurately extract nonlinear features from the response signals of the sensor array.

In the output for S1, the traditional BP model yielded an *RMSE* of 29.0586 and a coefficient of determination (*R*^2^) of 0.9394. The Sledge Dog Optimizer (SDO-BP) demonstrated the most robust optimization capability, reducing the *RMSE* to 16.7349 and elevating the *R*^2^ to 0.9798. This proves that the SDO algorithm, by simulating the collaborative search mechanism of sledge dog teams, can more effectively locate the global optimal weights within a vast search space, thereby significantly improving the fitting precision of the model for large-amplitude signals.

In the output for S3 (NH_3_ sensor), SFOA-BP achieved the highest goodness of fit among all models (*R*^2^ = 0.99977), with an *RMSE* of only 0.49447—a reduction of approximately 76% compared to the traditional BP network. This indicates that the SFOA algorithm possesses exceptional local exploitation capability when processing sensor signals with specific nonlinear characteristics, enabling near-perfect concentration inversion.

Predicting the output for S4 (H_2_ sensor) presents a greater challenge, as evidenced by the traditional BP’s *RMSE* of 13.7232. SDO-BP once again demonstrated its outstanding robustness, reducing the *RMSE* to 2.17—a performance improvement of 84.2%. Its exceptionally high *R*^2^ (0.9997) signifies that the algorithm has successfully addressed the cross-sensitivity issues of the sensor array under complex gas compositions.

### 3.5. Comparative Analysis with Related Multi-Gas Sensing Studies

To further validate the superiority of the proposed SDO-BP model, a comprehensive comparison is conducted between this study and recent representative works in the field of multi-gas quantification. As summarized in Table 7, the performance of the SDO-optimized network is compared with models such as DNN_TS, SVM/MLP, and LDNN.

While the cited studies demonstrate high precision for VOCs or specific inorganic gases, our work focuses on a complex 4-component industrial gas system (H_2_-CO-NH_3_-NO_2_) that exhibits severe cross-sensitivity. Compared to the DNN and SVM-based approaches in Ref [2] and Ref [3], the SDO-BP model in this study achieves a higher coefficient of determination (*R*^2^ > 0.99). This is attributed to the SDO algorithm’s collaborative search mechanism, which effectively prevents the BP network from falling into local optima when handling the 85-dimensional parameter space. The significant reduction in H_2_ *RMSE* (an 84.2% improvement over the baseline BP) demonstrates the robustness of our system in industrial safety monitoring scenarios.

In summary, the SDO-BP (Sledge Dog Optimizer) demonstrated the most comprehensive performance in this set of experiments. It not only achieved the optimal results for outputs S1 and S4 but also maintained exceptionally high precision and remarkably low *MAPE* across all other dimensions. The experimental results prove that the SDO-BP algorithm can significantly mitigate the inherent tendency of traditional BP networks to become trapped in local optima, thereby enhancing the reliability of mixed gas detection.

## 4. Conclusions

In this study, to address the challenges of cross-sensitivity and nonlinear coupling caused by the coexistence of multi-component gases (H_2_, CO, NH_3_, NO_2_) in industrial environments, a flow-through quantitative detection system based on a MEMS sensor array was developed. A concentration inversion scheme combining the steady-state peak sampling method with a swarm intelligence-optimized BP neural network was proposed.

By extracting steady-state voltage features at the 240th second of the gas-flow cycle, effective dimensionality reduction of high-dimensional time-series data was achieved, thereby eliminating interference from transient fluctuations. Experimental results demonstrate that the introduction of intelligent optimization algorithms significantly enhances the model’s performance in decoupling the “one-gas–multiple-responses” phenomenon. Notably, the Sledge Dog Optimizer-based BP model (SDO-BP) exhibited superior global optimization capability and robustness, achieving high-precision quantitative inversion for all gas components. This research successfully overcomes the signal coupling bottlenecks in complex atmospheres and provides reliable and accurate technical support for safety monitoring in industrial environments.

Although the proposed SDO-BP model demonstrates high precision in multi-component gas detection, certain environmental variables were not fully accounted for in this stage of the study. Metal Oxide Semiconductor (MOS) and MEMS gas sensors are intrinsically sensitive to variations in ambient temperature and relative humidity. In our current experimental setup, we mitigated these effects by employing a flow-through detection scheme and maintaining a relatively stable laboratory environment. However, in real-world industrial deployments, extreme temperature fluctuations and high humidity can significantly interfere with the charge exchange process on the sensor surface, potentially leading to increased prediction errors.

Future investigations will focus on: integrating temperature and humidity sensors into the acquisition system for real-time hardware compensation; conducting long-term stability tests in diverse industrial field environments to enhance the robustness of the quantitative inversion model; and expanding the sensor array with additional elements to improve selectivity and better adapt to complex, multi-component gas environments.

## Figures and Tables

**Table 1:** The sensor used.

Sensor	Manufacturer	Detection Range	Gases/Sensitivity
CM-B107S	Ningbo CSMicroSensor Co., Ltd. (Ningbo, China)	0.1–20,000 ppm	Ethanol, Ammonia, Carbon Monoxide, Methane
CM-B108S	Ningbo CSMicroSensor Co., Ltd. (Ningbo, China)	1–1000 ppm	Carbon Monoxide, Hydrogen Sulfide, Ammonia, Formaldehyde, Xylene
CM-B105S	Ningbo CSMicroSensor Co., Ltd. (Ningbo, China)	0.05–100 ppm	Ammonia, Hydrogen Sulfide, Carbon Monoxide, Formaldehyde, Xylene
GM-102	Zhengzhou Winsen Co., Ltd. (Zhengzhou, China)	0.1–10 ppm	Nitrogen Dioxide

**Table 2:** Concentration test points of the gas sensor array.

	1	2	3	4	5	6	7
CO (ppm)	100	200	350	400	450	500	550
NO_2_ (ppm)	1	2	2.5	3.5	5	7	8
NH_3_ (ppm)	5	10	20	30	50	75	90
H_2_ (ppm)	10	50	100	150	200	300	360

**Table 3:** Neural Network Configuration.

Category	Parameter	Value/Setting
Network structure	Input Neurons	4
	Hidden Layers	1
	Hidden Neurons	9
	Output Neurons	4
Transfer function	Activation Function	logsig
	Output Function	purelin
Training parameters	Optimization Method	trainlm
	Number of Epochs	1000
	Learning Rate	0.1
	Goal	1 × 10^−5^
	Momentum Factor	0.01

**Table 4:** Swarm Intelligence Parameters.

Algorithm	Population Size (N)	Number of Iterations (T)	Search Space Dimension (D)	Coefficients
AOO	10	20	85	$c={\left(1-t∕T\right)}^{3}$
ALA	30	20	85	$\theta =2\mathrm{atan}\left(1-t∕T\right)$
SFOA	30	20	85	$\omega =\left(\pi ∕2\right)\left(t∕T\right)$ $,\;k=0.2\sin\left(\pi ∕2-\omega \right)$
SDO	30	20	85	$\omega_{1}=1-t∕T$ $,\;p_{1}=1-\sqrt{t∕T}+0.5\left|y\right|\left(t∕T\right)$

**Table 5:** Representative Experimental Samples of Gas Concentrations and Sensor Array Responses.

	CO (ppm)	NO_2_ (ppm)	NH_3_ (ppm)	H_2_ (ppm)	S1 (mv)	S2 (mv)	S3 (mv)	S4 (mv)
1	100	1	5	10	162	2924	173	51
2	100	2	10	50	165	3062	319	233
3	100	2.5	20	100	166	3135	628	464
4	100	3.5	30	150	167	3248	931	687
5	100	5	50	200	169	3411	1523	911
…	…	…	…	…	…	…	…	…
39	830	2926	2778	711	500	1	90	150
40	809	3066	203	943	500	2	5	200
41	811	3126	354	1407	500	2.5	10	300
42	814	3237	663	1687	500	3.5	20	360
43	817	3422	969	58	500	5	30	10
44	820	3657	1577	251	500	7	50	50
45	823	3779	2315	486	500	8	75	100
46	911	823	2791	715	550	1	90	150
47	892	3087	207	949	550	2	5	200
…	…	…	…	…	…	…	…	…
91	450	7	20	360	732	3650	649	1686
92	450	8	30	10	734	3770	953	58
93	500	1	50	50	824	2925	1561	249
94	500	2	75	100	826	3065	2303	488
95	550	2.5	90	150	907	3140	2779	713

**Table 6:** Quantitative Performance Comparison of Different Models Across Four Target Gases.

Indicator	Models	CO	NO_2_	NH_3_	H_2_
*R* ^2^	BP	0.93942	0.98466	0.99575	0.98609
	ALABP	0.95758	0.99660	0.99955	0.99969
	AOOBP	0.95795	0.99867	0.99893	0.99852
	SFOABP	0.96683	0.99634	0.99977	0.99937
	SDOBP	0.97981	0.99657	0.99943	0.99970
*RMSE*	BP	29.0586	0.33473	2.07540	13.7232
	ALABP	23.1720	0.16259	0.68577	2.1914
	AOOBP	22.7074	0.10133	1.06710	4.9328
	SFOABP	19.9669	0.16323	0.49447	3.1280
	SDOBP	16.7349	0.16225	0.77376	2.1700
*MAPE*	BP	0.046659%	0.118210%	0.061620%	0.262330%
	ALABP	0.064859%	0.072245%	0.044304%	0.061200%
	AOOBP	0.050986%	0.042727%	0.056371%	0.089961%
	SFOABP	0.066506%	0.077156%	0.040176%	0.052470%
	SDOBP	0.037173%	0.070429%	0.047745%	0.049067%

**Table 7:** Comparison of the proposed system with related multi-gas sensing studies.

Source	Sensing Technology	Target Components	Optimization	Key Performance Metrics
Ref [2]	SnO_2_ Hollow-spheres Array	Various VOCs	DNN_TS	*Error*: 4.75%
Ref [3]	17 MOS Sensor Array	Fungal Metabolites	SVM/MLP	*R*^2^: 0.975
Ref [4]	4-channel Semiconductor Array	NO_2_, NH_3_, CH_4_, CO_2_	LDNN + AEN	*MRE*: < 1.17%
This Work	MEMS Sensor Array	H_2_, CO, NH_3_, NO_2_	SDO-BP	*R*^2^: 0.999, H_2_ *RMSE*: 2.17

## Data Availability

The original contributions presented in this study are included in the article. Further inquiries can be directed to the corresponding author.
